# Placenta accreta in the department of gynaecology and obstetrics in Rabat, Morocco: case series and review of the literature

**DOI:** 10.11604/pamj.2019.33.86.17700

**Published:** 2019-06-06

**Authors:** Aziz Slaoui, Sarah Talib, Anass Nah, Kamal El Moussaoui, Intissar Benzina, Najia Zeraidi, Aziz Baydada, Aicha Kharbach

**Affiliations:** 1 Gynaecology-Obstetrics and Endocrinology Department, Maternity Souissi, University Hospital Center IBN SINA, University Mohammed V, Rabat, Morocco; 2 Gynaecology-Obstetrics and Endoscopy Department, Maternity Souissi, University Hospital Center IBN SINA, University Mohammed V, Rabat, Morocco

**Keywords:** Abnormal placentation, accreta, percreta, indicated preterm delivery, caesarean hysterectomy

## Abstract

Placenta accreta spectrum disorders is a rare pathology but the incidence has not stopped to increase in recent years. The purpose of our work was the analysis of the epidemiological profile of our patients, the circumstances of diagnosis, the interest of paraclinical explorations in antenatal diagnosis and the evaluation of the evolutionary profile. We hereby report a case series spread over a period of one year from 01/01/2015 to 01/01/2016 at the Gynaecology-Obstetrics department of the University Hospital Center IBN SINA of Rabat where we identified six cases of placenta accreta. We selected patients whose diagnosis was confirmed clinically and histologically. The major risk factors identified were a history of placenta previa, previous caesarean section, advanced maternal age, multiparity. 2D ultrasound and magnetic resonance imaging (MRI) allowed us to strongly suspect the presence of a placenta accreta in a pregnant woman with risk factor(s) but the diagnosis of certainty was always histological. Placenta accreta spectrum disorders were associated with a high risk of severe postpartum hemorrhage, serious comorbidities, and maternal death. Leaving the placenta in situ was an option for women who desire to preserve their fertility and agree to continuous long-term monitoring in centers with adequate expertise but a primary elective caesarean hysterectomy was the safest and most practical option. Placenta accreta spectrum disorders is an uncommon pathology that must be systematically sought in a parturient with risk factors, to avoid serious complications. In light of the latest International Federation of Gynecology and Obstetrics (FIGO) recommendations of 2018, a review of the literature and finally the experience of our center, we propose a course of action according to whether the diagnosis of the placenta is antenatal or perpartum.

## Introduction

The 2018 International Federation of Gynecology and Obstetrics (FIGO) consensus described the placenta accreta spectrum disorders by three categories: first, the adherent placenta accreta when the villi simply adhere to the myometrium; second the placenta increta, when the villi invade the myometrium; and third the placenta percreta, when villi invade the full thickness of the myometrium including the uterine serosa and sometimes adjacent pelvic organs [1]. Its incidence has been rising in recent years and this appears to correlate with the increase of caesarean section rates. Major risk factors are a history of placenta previa, previous caesarean section, advanced maternal age, multiparity and a history of endo-uterine maneuvers [2]. The diagnosis may be antenatal, based primarily on obstetrical ultrasound, Doppler ultrasound and magnetic resonance imaging (MRI), but may be discovered after a failed delivery. The diagnosis of certainty is histological; it is mainly posed per-partum in front of the absence of cleavage zone between the placenta and the myometrium thus making delivery difficult or impossible [3]. The first-line treatment has long been hysterectomy, but current advances in surgical and hemostatic techniques have improved the prognosis in postpartum hemorrhage allowing us to experiment a conservative treatment. This conservative treatment must be applied with caution and in a suitable infrastructure and would allow the preservation of subsequent fertility as well as the reduction of morbidity and maternal-fetal mortality [3]. Placenta accreta spectrum disorders is associated with a high risk of severe postpartum hemorrhage, serious comorbidities, and maternal death. According to World Health Organization, the main cause of maternal death is severe bleeding (mostly bleeding after child birth) which is why these disorders have become a public health problem [4]. It is therefore essential that obstetricians be up-to-date to properly manage patients by following the latest recommendations from learned societies.

## Methods

We hereby report a case series spread over a period of one year from 01/01/2015 to 01/01/2016 at the Gynaecology-Obstetrics Department of the University Hospital Center IBN SINA of Rabat where we identified six cases of placenta accreta. Since all cases were not diagnosed before birth, patients were identified retrospectively by hand-searching the register of obstetrics and gynaecology department from 1^st^ January 2015 through 1^st^ January 2016 for all patients where diagnosis of placenta accreta was mentioned. We subsequently decided to include cases where the diagnosis was confirmed histologically and we excluded cases where the degree of invasion was not determined. Examination of the clinical record was used to determine if placenta accreta was suspected before delivery but also to recover all the maternal variables including number of pregnancies, number of previous cesarean deliveries, history of previous uterine curettage, gestational age, concurrent placenta previa, history of preeclampsia, eclampsia, chronic hypertension, gestational diabetes, diabetes mellitus, pulmonary disease, cardiac disease, maternal mortality and Apgar scores at 1, 5 and 10 minutes. The paraclinical examinations used could be found in the medical file. The operative report allowed us to recover the surgery performed but also the type of anesthesia used. Intraoperative blood loss was obtained from the anesthesia record and was based on the amount of blood in the suction reservoir plus the estimated blood in surgical pads (100mL for each soaked surgical pad, 100mL for each five partially soaked surgical pads and 10mL for each soaked surgical gauze). The number of units and type of blood components (RBC, FFP or platelets) used intraoperatively and 48 hours postoperatively were also recorded. Intraoperative photographs were retrieved from the database of our department. Given the small number of cases, a statistical analysis did not seem relevant. We then opted for the presentation of each case in the form of detailed clinical observations as well as their therapeutic management.

**Ethics approval and consent to participate:** ethics approval has been obtained to proceed with the current study. Written informed consent was obtained from the patient for participation in this publication.

## Results

**First case:** 28-years-old patient with no notable pathological antecedents, gravida 2 para 2 with one living child. G1: caesarean section performed in Tangier for a small pelvis 4 years ago, undetermined birth weight, good psychomotor development. G2: caesarean section performed for haemorrhagic placenta previa at 30 weeks of amenorrhea performed in Tangier, then the patient was referred to our formation after the discovery of a placenta percreta which is left in place, cord cut to rat. The admission examination found a patient conscious, afebrile, blood pressure at 8/5, pulse rate at 100 beats per minute, discoloured conjunctiva. The obstetric examination found a non-firm and non-globular uterus and red bleeding of average abundance. The patient was put in condition, blood tests were requested including CBC, PT, aPTT, fibrinogen blood test, blood typing, CRP blood test, CBEU and HVS. At the same time the patient received 60 IU of syntocynon, 5cp of Cytotec (200 micrograms of misoprostol) intra-rectal then transfused by 2 packed red blood cells with antibiotic therapy based on cephalosporin 3rd generation. An MRI was performed urgently, doing the diagnosis of placenta percreta invading the posterior surface of the bladder. After stabilization of the hemodynamic state and drying up of bleeding, the patient received methotrexate. With a very close monitoring of the infectious balance and that of the haemostasis blood tests. Ultrasonographic controls did not mention a large change in placental size with persistent intermittent bleeding episodes for which the patient was transfused again. A decision of embolization in this young patient was taken and on the fifteenth day of her hospitalization this embolization was carried out. The evolution was favorable; the patient left the hospital after 28 days.

**Figure 1:**
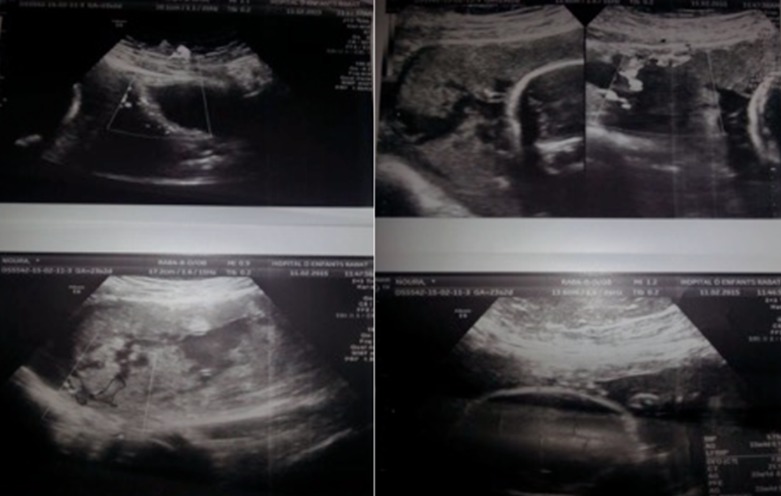
Doppler ultrasound showing vascularization crossing most of the thickness of the myometrium: placenta accreta

**Figure 2:**
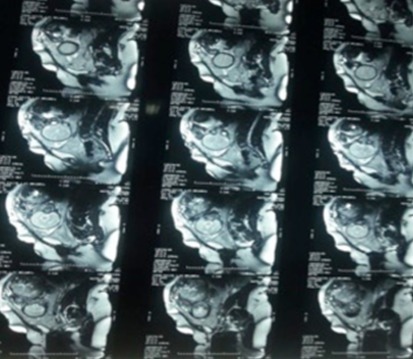
MRI images suggestive of placenta percreta

**Second case:** 36-years-old patient with no notable pathological history, gravida 4 para 4 with 2 live children. G1: caesarean section performed in 2002 for acute foetal distress at the beginning of labor with a new-born who died at day 1 of life in a context of infection. G2: caesarean section performed in 2005 for unspecified cause, undetermined birth weight, good psychomotor development. G3: caesarean section performed for double scarred uterus, undetermined birth weight, good psychomotor development. G4: fourth pregnancy whose prenatal follow-up objectified placenta accreta. An ultrasound performed at the 24 weeks of amenorrhea showed a posterior placenta accreta with presence of vascular gaps with venous flow, myometrial thinning and an intense abnormal vascularization in the serosa (Figure 1). An MRI requested 2 months later showed a percreta placenta with invasion of the bladder wall: irregular aspect of the postero-superior wall of the bladder with disappearance of the interface separating it from the myometrium over a wide area of 6cm (Figure 2). The patient was hospitalized at 32 weeks of amenorrhea. She underwent close monitoring, her hemostasis blood tests were correct, twice-daily fetal heart rate records were normal. In collaboration with the urologists' staff, it was decided to mount a double-J probe on the eve of the planned cesarean section at 38 weeks of amenorrhea. Under general anesthesia, the old umbilical medial laparotomy scar was taken up. Huge musculo-aponevrotico-uterine adhesions were found. The incision was then enlarged in supraumbilical. After release of adhesions a corporal hysterotomy was realized to allow the podalic extraction of a female newborn Apgar score at 10 and birth weight 3000g. Artificial delivery was possible with an adherent area partially on the inferior and anterior surface of the uterus. The bleeding that came from it was controlled by X-stitches and a triple ligation with uterine artery ligation, round ligament ligation, utero-ovarian ligament ligation was subsequently achieved. Blood loss was estimated at 3000cc. The patient received a total of 9 packed red cells and 7 fresh frozen plates iso group iso Rhesus. In immediate postoperative the patient was hemodynamically stable, with ensured hemostasis and a firm and globular uterus. 20h after her admission to the intensive care unit, she presented unstable blood pressure, maintained by an adrenaline infusion then a cardiac arrhythmia type ventricular tachycardia jugulated by lidocaine and finally an irreversible ventricular flutter despite electrocardiac shock and lidocaine. The patient died.

**Third case:** 36-years-old patient with no significant pathological history, gravida 3 para 3 with 2 live children. G1: caesarean section performed for transverse fetal lie, birth weight 3200g, good psychomotor development. G2: vaginal delivery, birth weight 2800g, good psychomotor development. G3: third pregnancy followed in Kenitra for low-lying placenta for which she was hospitalized at 20 weeks of amenorrhea and transfused by 4 red blood cells then transferred in our structure for additional medical care. The admission examination found a normotensive patient with fever at 38.2ºC, conjunctiva slightly discolored. The obstetrical examination found uterine height at 16cm with minimal reddish bleeding. An ultrasound was performed and showed a progressive monofetalous pregnancy, fetal biometry at 20 weeks of amenorrhea with hydramnios and a low-lying placenta. The patient was hospitalized for medical supervision and blood tests were requested including CBC, PT, aPTT, fibrinogen blood test, blood ionogram test, CRP blood test, blood typing and of course a CBEU and HVS. Twenty four hours of her hospitalization, the parturient presented a massive uterine haemorrhage for which a cesarean section for maternal rescue was indicated. This allowed the extraction of a male newborn, Apgar score at 1/10, birth weight at 650g, died at the first hour of life. At the exploration, the artificial delivery was impossible because of a placenta acreta, for which we realized a subtotal interadnexal hysterectomy. The blood loss was estimated at 3500cc, the patient received 12 red blood cells and 10 fresh frozen plates iso group iso Rhesus. Six hours after the caesarean section, the patient presented an abdominopelvic effusion and a tachycardia at 140 bpm for which she was resumed. At exploration, we found a hematoma of the broad ligament. After having ensured hemostasis, the patient stayed in the intensive care unit. The evolution was favorable, the patient left the hospital after 12 days.

**Figure 3:**
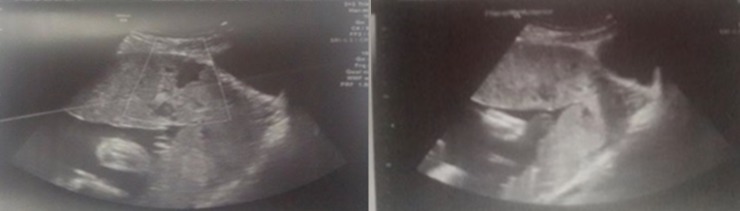
Ultrasound images suggestive of placenta percreta

**Figure 4:**
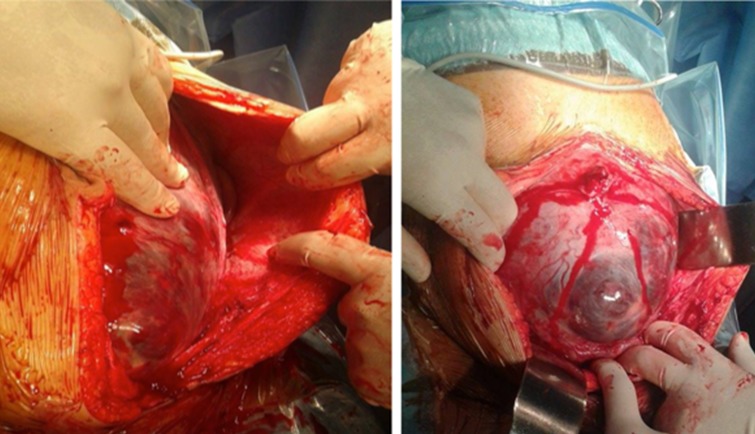
Images showing placenta accreta with macroscopic invasion of the myometer

**Fourth case:** 32-years-old patient with no pathological history, gravida 3 para 3 with 2 live children. G1: caesarean section performed 8 years ago for undetermined cause, birth weight unknown, good psychomotor development. G2: caesarean section performed 4 years ago for acute fetal distress at the beginning of labor, birth weight 3700g, good psychomotor development. G3: third pregnancy estimated at 30 weeks of amenorrhea at the time of her admission in our structure. Admitted through the emergency department for metrorrhagia of the third trimester of pregnancy, the examination found a normotensive patient with apyrexis, slightly discolored conjunctiva. The obstetrical examination found a minimal reddish bleeding. Doppler ultrasound showed an active monofetalous pregnancy in cephalic presentation, fetal biometry at 29 weeks of amenorrhea with hydramnios and a low-lying placenta percreta (Figure 3). An MRI then performed supported the diagnosis of placenta percreta. The patient was hospitalized with close monitoring of uterine contractions, fetal heart sounds and bleeding as well as biological status, and twice-daily fetal heart rate records. The patient with no desire for fertility accepted our proposal to conduct a non-conservative treatment. At 38 weeks of amenorrhea a scheduled cesarean section was performed which allowed the cephalic extraction of a female newborn, Apgar score at 10, birth weight 3150g. The exploration found a placenta accreta with intact bladder (Figure 4). A subtotal inter-adnexal hysterectomy was then performed. Blood loss was estimated at 2000cc, the patient received 6 red blood cells and 4 fresh frozen plates iso group iso Rhesus. The evolution was favorable, she left the hospital after 10 days.

**Fifth case:** 36-years-old patient with no significant pathological history, gravida 4 para 4 with 3 live children. G1: caesarean section 10 years ago for undetermined cause, birth weight unknown, good psychomotor development. G2: caesarean section 6 years ago for acute fetal distress, birth weight unknown, good psychomotor development. G3: caesarean section 3 years ago for double scarred uterus, birth weight unknown, good psychomotor development. G4: fourth pregnancy estimated at 34 weeks of amenorrhea at the time of her admission in our structure. She was hospitalized for macroscopic hematuria. Doppler ultrasound objectified an active monofetalous pregnancy in cephalic presentation, fetal biometry at 34 weeks of amenorrhea, normal amount of amniotic liquid and a low-lying placenta percreta with vascularization passing through the uterine thickness into the bladder. An MRI confirmed the suspicion of placenta percreta previa with bladder invasion without dilatation of ureters and pyelocalical cavities. The patient underwent close monitoring of uterine contractions, fetal heart sounds and bleeding as well as biological status, and twice-daily fetal heart rate records. In collaboration with the urologists' staff it was decided to mount a double-J probe on the eve of the planned cesarean section at 38 weeks of amenorrhea. The scheduled cesarean section was then performed and allowed the birth of a male newborn, Apgar score at 10, birth weight 3450g. At the exploration, the artificial delivery was made impossible because of the percreta placenta, an interadnexal subtotal hysterectomy was performed with a partial cystectomy (removal of the bladder dome respecting the trigone). The blood loss was estimated at 1500cc, the patient received 5 red blood cells and 3 fresh frozen plates iso group iso Rhesus. The follow-up was simple and the postoperative intravenous urography was normal. After ten days of favorable evolution, she left the hospital.

**Sixth case:** 36-years-old patient with no significant pathological history, gravida 4 para 4 with 3 live children. G1: caesarean section 12 years ago for a breech presentation, birth weight unknown, good psychomotor development. G2: caesarean section 8 years ago for acute fetal distress, birth weight unknown, good psychomotor development. G3: caesarean section 5 years ago for double scarred uterus, birth weight unknown, good psychomotor development. G4: fourth pregnancy estimated at 38 weeks of amenorrhea at the time of her admission in our structure. She was admitted for metrorrhagia of the third trimester of pregnancy associated with uterine contractions, the examination had found a conscious patient, hemodynamically stable with discolored conjunctiva. The uterine height was 31 cm, the fetal heart sounds were present and regular, uterine contractions were coming in regular intervals. Speculum examination revealed massive reddish bleeding from the endocervix. The patient was directly referred to the operating room for an emergency caesarean section, which allowed the birth of a male new-born, Apgar score at 10, birth weight 3200g. The placenta was attached firmly to the uterine wall making unsuccessful extirpative method and therefore artificial delivery was impossible. A hysterorraphy was then performed, followed by a subtotal interadnexal hysterectomy after ligation of the hypogastric arteries. The blood loss was estimated at 1500cc. The patient was transferred to intensive care and transfused with 4 red blood cells. The evolution was favorable and the patient left the hospital after 12 days.

## Discussion

### Epidemiology

**Incidence:** it is still difficult to accurately assess the frequency of placenta accreta. Indeed, their incidence varies considerably according to the studies, the period studied and also according to the definition given to placenta accreta: some teams only accept the diagnosis in case of pathological evidence [5]. In addition, in case of conservative treatment it is always difficult to provide this histological evidence. During the period of our study i.e. one year, we have confirmed six cases of placenta accreta on 34947 births in the maternity Souissi Rabat which gives us an incidence rate of 1/5824 or 0.017%. Our incidence rate remains very low compared to data from the literature. In 2017, the first national and binational case-control study of placenta accreta in Australia and New Zealand found an incidence rate of 44.2/100000 women given birth or 0.0442% [6]. The most cited epidemiological study is that of Miller *et al.* who found in the United States over a period of 10 years (1985-1994) 62 placentas accreta on 155 670 births with an incidence rate of 1/2 510 births or 0.0398% [5]. This lower incidence in Morocco can be explained by the fact that screening is more effective in developed countries than in low- and middle-income countries. The only certainty is that the incidence of placenta accreta has increased dramatically in a few decades, and it was shown that this was likely correlated to the increasing rate of cesarean delivery [7]. Furthermore, the widespread use of ultrasound including looking for signs of placental accretisation in patients with a scarred uterus, alone represents an essential factor in the increased incidence of this disease. This trend is not expected to change in the coming years given the current obstetrical practices regarding cesarean indications such as breech presentation, twin pregnancies and scarred uteri. This is why it is important to identify women at risk in order to establish an appropriate management.

**Risk factors:** several risk factors have been reported in the literature but the one that comes up most often is the occurrence of caesarean section. In a 20-year retrospective study, Wu *et al.* [2] studied the risk factors for placenta accreta in a population of 121 cases compared to a control population. It is evident from these data that one of the major risk factors for placenta accreta is cesarean section, especially their number. Indeed they showed that the rate of occurrence of this anomaly increases with the number of previous cesarean sections, multiplying by 3 or 4 when there are 2 or more antecedents compared to a single caesarean section [2]. The second risk factor found in the literature is placenta praevia. Miller *et al.* [5] reported an incidence rate of 1/68 000 births or 0.0015% in the absence of any risk factor to nearly 10% of births in patients with a low-lying placenta. Indeed, the low insertion of the placenta exposes to an invasion accreta, because of the topography of the placental insertion in a place (uterine isthmus and internal cervix orifice) where the functional quality of the decidua is lacking [5]. In addition, the authors of a recent study warn practitioners that within the patients with placenta previa: placental attachment to the incision site and full placenta previa further increase the risk of accretion [8]. Other less significant risk factors could be identified including: advanced maternal age, multiparity, endometritis or any endo-uterine gesture favoring an abnormality in the reconstruction of the endometrium, such as curettage, myomectomies or metroplasty [9]. Moreover, in a study published by Alanis *et al*. it was found that among the 15% of women who had a subsequent pregnancy after a placenta accreta, 18% recidivated [10]. The history of placenta accreta can therefore be considered as a risk factor in the occurrence of this pathology, especially since the risk factors present in the previous pregnancy are always present during the subsequent pregnancy. Due to the insufficient number of cases in our study, an epidemiological analysis wouldn't be relevant but it would be interesting to look for these risk factors in our sample. In fact these results match perfectly our study where the average age of our patients was 34 years of which 66% are older than 35 years. The main obstetrical antecedent represented by the scarred uterus found in 100% of cases with the presence of placenta previa found in 50% of cases.

**Diagnosis:** schematically, the diagnosis of placenta accreta can be made, either in the prenatal period mainly in the context of placenta previa implanted on a caesarean section scar, or unfortunately too often during serious complications of delivery. In our study, Doppler ultrasound made possible the antenatal diagnosis in 3 of our 6 patients. An MRI was performed to confirm the ultrasound images. This low proportion of antenatal diagnosis is in agreement with the studies, in particular that of Clouqueur *et al.* [3]: only 6 of 21 patients have benefited from an antenatal diagnosis. That was also the case in O'brien *et al.'s* study [11], where only 50% of the 109 cases of placenta percreta were diagnosed prenatally. This difficulty in diagnosing placentas accreta during the prenatal period can be explained by the high frequency of risk factors. In point of fact, it is difficult to perform an effective screening on a large population with this type of complementary examinations. The diagnosis is made mainly in the presence of the association of placenta previa and a cicatricial uterus. In our series, concerning the patients who benefited from an antenatal diagnosis, 3 of 3 presented this association. In the Clouqeur *et al.* study [3] this association represents 5 out of 6 patients. From our results, it seems that Doppler ultrasound is effective to diagnose placenta accreta in a population at risk; this is consistent with the literature. Chou *et al.'s* study [12] of 64 cases of placenta accreta, evaluating the efficacy of Doppler ultrasound in the antenatal diagnosis of placenta accreta in a population at risk (previous caesarean section and placenta previa) found a good specificity (96.8%) and a good negative predictive value (95.3%).

### Ultrasound signs

**The presence of intra-placental lacunaes:** intraplacental lacunae were found in our 3 cases that benefited from a prenatal diagnosis. According to these results, the intraplacental deficiencies are thus a predictive sign of placenta accreta, which is in agreement with the study of Yang *et al.* [13], which finds a specificity of 78.6% and a sensitivity of 86.9% in the presence of gaps. It is thus considered as the most reliable sign in the diagnosis of placenta accreta. Finberg *et al.* [14] have established a classification in 4 stages: Stage 0: no lacunae. Stage 1: 1 to 3 small sized lacunas. Stage 2: 4 to 6 bigger lacunaes with irregularities. Stage 3: more than 6 lacunaes occupying all the placental thickness. Finger *et al.* [14] were able to show that the positive predictive value of this criterion increases with stage.

**Interruption of the hyperechoic zone at the interface of the uterine serosa and the bladder:** according to Palacios *et al.'s* study [15], the interruption of this limit is a specific but insensitive sign. Other authors found that some aspects of bulging towards the bladder may be visible and are predictive of a placenta accreta [16]. We found this interruption in our fourth patient where the diagnostic of placenta percreta was made.

**Thinning of the myometrium next to the bladder:** some authors such as Finberg *et al.* [14] showed that a thickness of less than 1 mm would be a predictive measure of accreta. This thinning was objectified in the doppler of our second patient.

**MRI signs:** given the limitations of the ultrasound examination on several points like posterior placental locations that are difficult to access, MRI has the advantage of being three-dimensional and provides greater anatomical topographic accuracy of the invasion and the extension to adjacent organs. In our study, we used MRI in 4 of our 6 patients to strongly support the diagnosis of accretisation but all of the placentas were in anterior position. Our results are then not comparable to the literature, which attributes to MRI, an important utility in cases of posterior placenta. Like Levine *et al.* [17] who compared MRI with Doppler ultrasound in 19 patients, concluding that MRI could be more effective only in posterior placental insertions. Indeed, the low number of MRIs in our series does not allow us to evaluate the diagnosis value according to the anterior or posterior nature of the placenta. However, the MRI allowed us to assess the degree of placental invasion. These results are in agreement with Maldijan *et al.'s* study [18], which concluded that MRI could visualize better the degree of placental invasion and thus identify better placentas percreta and their relationship to the bladder.

**Therapeutic management:** placenta accreta spectrum disorders is a high-risk situation for severe post-partum bleeding and its complications such as disseminated intravascular coagulation, haemostasis hysterectomy, surgical wounds of the ureters and the bladder, multi-organ failure or even maternal death; especially in the case of placenta percreta [11]. The choice of the management strategy depends mainly on the anatomical type of the placenta encountered and the subsequent desire for fertility of the patient. The treatment must be multidisciplinary. Obstetricians, radiologists, aenesthesits and sometimes urologists and visceralists work concomitantly [19, 20]. In our series, the main complication was the hemorrhagic shock that all our patients endured. The average packed RBCs per patient was 6.4 and the average number of FFP transfusion per patient was 4. The maternal mortality rate in our series was 17% (1 patient of 6) and the neonatal mortality rate was 0%. Maternal mortality is 7% for O'Brien *et al.* (8/109 patients) [11]. Fetal risks are also important because in the same series of O’Brien *et al.* there were 10 deaths (9%), including 6 for gestational age around 22 weeks of amenorrhea [11]. Conservative treatment was performed in two patients who have had an antenatal diagnosis: the first patient successfully underwent selective embolization after failure of methotrexate treatment and the second patient underwent triple ligation with uterine artery ligation, round ligament ligation and utero-ovarian ligament ligation but died 20 hours after her admission in the intensive care unit of a second hemorrhagic shock that could not be curbed. Non-conservative treatment with interadnexal subtotal hysterectomy was successfully performed in 4 patients, one of whom had an antenatal diagnosis but no longer had a desire for fertility. In light of the latest FIGO recommendations of 2018, a review of the literature and finally the experience of our center, we propose a course of action according to whether the diagnosis of the placenta is antenatal or perpartum [18-22].

**Antenatal diagnosis:** for patients wishing to have a future pregnancy, we opt for a conservative treatment with placement of preoperative arterial embolization probes, followed by scheduled caesarean section and, if possible, resection of trophoblastic tissue. Otherwise it will be left in place. It is completed by postoperative embolization. The extirpative method should be abandoned and the use of methotrexate is not indicated because of the lack of strong evidence of its efficacy. In case of complications related to conservative treatment such as secondary haemorrhage or infection: secondary hysterectomy is performed. For patients who do not wish to have a future pregnancy, we opt for a non-conservative treatment with a caesarean section followed by a total hysterectomy with placenta in situ (preferred over subtotal hysterectomy in cases of placenta previa increta or percreta) after ligation of the hypogastric arteries. If the operative risk is very important (lesions of neighboring organs), we opt for a conservative treatment.

**Figure 5:**
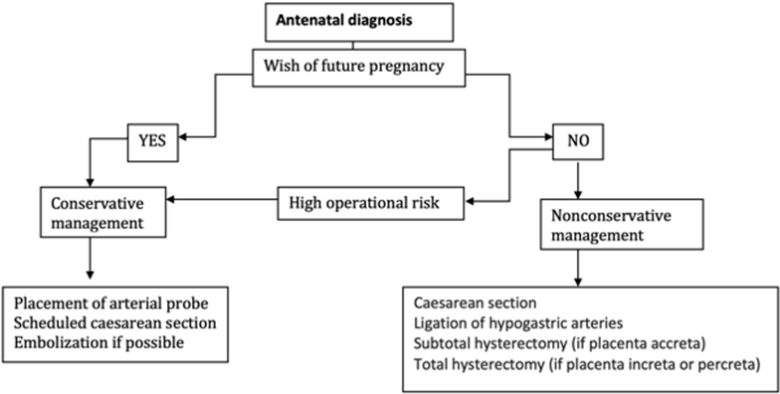
Tree decision diagram in case of placenta accreta's antenatal diagnosis

**Figure 6:**
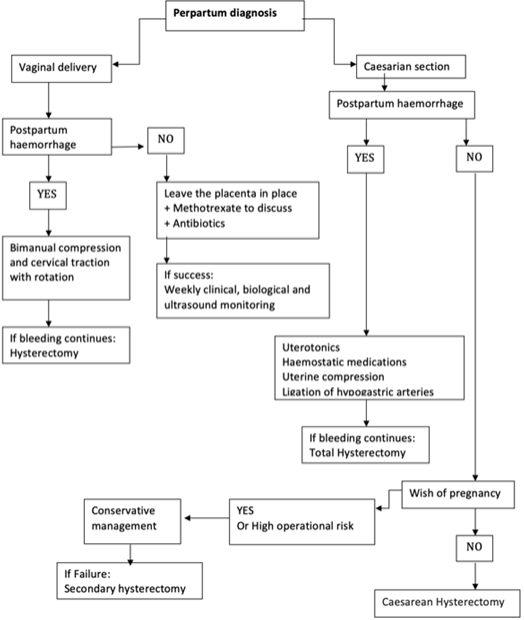
Tree decision diagram in case of placenta accreta's perpartum diagnosis

**Perpartum diagnosis:** in a bloodless context, we opt for a conservative or radical treatment depending on the patient's desire for subsequent fertility and operability. In case of severe haemorrhage of delivery, the various haemostatic techniques deployed in case of severe haemorrhage of delivery are started: uterotonic; hemostatic medication; uterine tamponade; ligation of hypogastric arteries. Finally, the failure of these measures imposes hemostasis hysterectomy. The two decision tree diagrams summarize the actions to be taken in both cases (Figure 5, Figure 6).

## Conclusion

Placenta accreta spectrum disorders is a rare pathology but the incidence has not stopped to increase in recent years. This trend is probably correlated to the increase of caesarean sections and certain risk factors such as the history of placenta previa, advanced maternal age and uterine surgery with mucosal erosion. This disease is burdened with heavy transfusion morbidity and a significant mortality. 2D ultrasound and MRI can strongly guide the presence or absence of an accreta in a pregnant woman with risk factor(s) of placenta accreta. The improvement of prenatal diagnosis allows an optimization of the care. According to the latest recommendations, leaving the placenta in situ is an option for women who desire to preserve their fertility and agree to continuous long-term monitoring in centers with adequate expertise. But a primary elective caesarean hysterectomy is the safest and most practical option for most low- and middle-income countries where diagnosis, follow-up and additional treatments are not available.

### What is known about this topic

- The placenta is a rare but increasing pathology mainly because of the increased number of caesareans;
- The presumptive diagnosis is ultrasonographic or at the MRI but it is only histology that confirms it;
- The choice of the management strategy depends mainly on the anatomical type of the placenta encountered and the subsequent desire for fertility of the patient.

### What this study adds

- The combination of a previous caesarean section with a low inserted placenta should always makes us suspect a placenta accreta
- A primary elective caesarean hysterectomy is the safest and most practical option for most low- and middle-income countries where diagnosis, follow-up, and additional treatments are not available.

## Competing interests

The authors declare no competing interests.
